# Evaluation of the new “C” parallel analyser

**DOI:** 10.1155/S1463924680000454

**Published:** 1980

**Authors:** Raija Puukka, M. Puukka

**Affiliations:** Department of Clinical Chemistry Oulu University Central Hospital Oulu 22 SF-90220 Finland


### Evaluation of the new "C" parallel analyser

**Raija Puukka and M. Puukka**

*Department of Clinical Chemistry, Oulu University Central Hospital, SF-90220 Oulu 22, Finland.*

### Introduction

The C Parallel Analyser (Kone Oy, Instrument Division, Espoo, Finland) is a compact, new clinical analyser for photometric equilibrium and kinetic analyses. The instrument consists of a 24-channel parallel photometer, a control console with numeric display, an alphanumeric printer and a microcomputer which controls the analyser and takes care of automatic blanking and calibration. Reaction mixtures are prepared off line in additional system modules. Measurements are made in blocks of 24 individually thermostated cuvettes which are manually introduced into the analyser measurement head. The same principle of parallel handling of the samples simultaneously has been used in a bigger parallel analyser, the System Olli 3000 [1].

The instrument has been evaluated for the clinical assay of seven tests which were selected to represent different types of chemical determinations [2]. The tests evaluated and their respective methods were as follows: alkaline phosphatase (ALP, EC 3.1.3.1; kinetic), aspartate aminotransferase (AST, EC 2.6.1.1; kinetic), alpha-amylase (EC 3.2.1.1; chromogenic), albumin (equilibrium), cholesterol (enzymatic equilibrium), creatinine (nonenzymatic kinetic) and triglycerides (enzymatic kinetic). The results indicate that the analyser is fast and versatile, and gives precise results comparable with those obtained by methods routinely used in the authors' laboratory.

### Materials and methods

Serum ALP and AST activities were determined according to the method recommended by the Committee on Enzymes of the Scandinavian Society for Clinical Chemistry and Clinical Physiology [3]. Both activities were measured routinely with the LKB 8600 reaction rate analyser (LKB-Produkter AB, Bromma, Sweden) and with the C analyser. Using the C analyser, the measurement was started within 1 min of adding the starting reagent. Reagents for ALP determination were obtained from Orion-Yhtymä Oy (Helsinki, Finland) and for AST determination from Medix Oy (Kauniainen, Finland).

Serum alpha-amylase activities were manually determined with a Phadebas Amylase test (Pharmacia Diagnostics AB, Uppsala, Sweden) using the procedure developed by the manufacturer, and with the C analyser using the procedure originally developed for the System Olli 3000 analyser [4]. The manual procedure was carried out using normal amylase tablets (batch no CH 1141). The analyser procedure used tablets which were half the weight of normal tablets (batch no RLFA 770 404, Pharmacia Diagnostics AB).

Serum albumin, cholesterol and creatinine determinations were carried out routinely with the SMAC (Technicon Instruments Corp, Tarrytown, New York, USA) using the original Technicon methods. (Technicon method no SG4-0030PG5 (bromocresol green), SG4-0026PH5 (direct Lieberman – Burchard) and SG4-0011PJ4 (alkaline picrate, equilibrium), respectively).

With the C analyser, serum albumin determinations were made using the bromocresol green dye-binding method, 600μl of 0.9% NaCl and 10μl of serum sample were pipetted into the cuvettes in the measuring block, and 800μl of bromocresol green reagent (General Diagnostics AB, Division of Warner-Lambert Co, Morris Plains, New Jersey, USA) was added simultaneously using the dispenser. The measurement was carried out 1 min after addition of the dye-reagent.

Serum cholesterol concentrations were determined enzymatically with the C analyser using a test kit "Test-Combination Cholesterol (CHOD-PAP)" (no 187 313, Boehringer Mannheim, Mannheim, West-Germany) as directed by the manufacturer. Calibration was performed using 7.5 mmol/l of aqueous cholesterol standard (Orion-Yhtymä Oy).

Serum creatinine concentrations were determined kinetically with the C analyser using a "Mercotest, Creatinine" test kit (no 3384, E Merck AB, Darmstadt, West-Germany). Reagents were prepared as directed by the manufacturer and the following procedure was used: 1.0 ml of a picric acid reagent and 100μl of serum sample were pipetted into the cuvettes in the measurement block. The block was then shaken and incubated for 15 min at 37°C. Measurement was started within 1 min 30 sec of adding 100μl of the buffer solution to the cuvettes with the dispenser. The measurement time was 2 min.

Serum triglyceride concentrations were routinely determined enzymatically by an equilibrium method using a "Triglycerides, fully enzymatic" test kit (no 126 039, Boehringer Mannheim) as described earlier [5]. With the C analyser, triglyceride concentrations were determined kinetically using a test kit – "Triglycerides, fully enzymatic kinetic UV-method" (no 244 473, Boehringer Mannheim). The procedure was similar to that proposed for the LKB reaction rate analyser by the manufacturer except that the measurements were started within 30 sec of adding the glycerol kinase reagent. Calibration was performed using 2.0 mmol/l of aqueous glycerol standard.

### Apparatus

The C Parallel Analyser consists of a 24-channel parallel photometer (with max. 12 interference filters covering the range 340-800 nm, absorbance range 0.000-2.000, bandwidth 8 nm in UV-filters and 10-15 nm in visible range

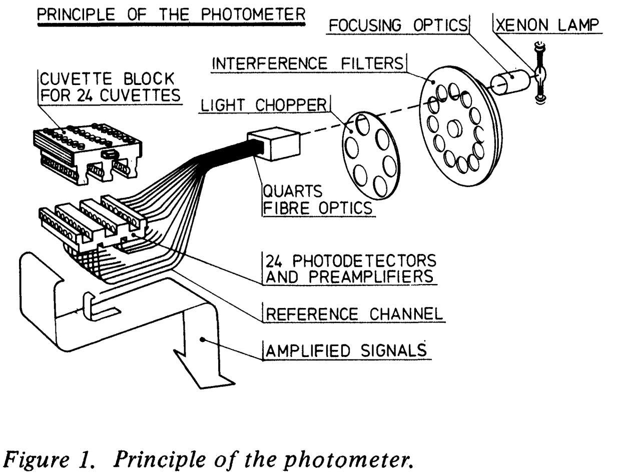

*Figure 1. Principle of the photometer.*

filters). It includes a control console with numeric display, an alphanumeric thermal printer and a microcomputer (8-bit). The analyser is designed to perform both equilibrium and kinetic determinations in batches of 24 (or less) samples; the minimum end volume is 500µl. Light from a single source (Xenon-lamp) is supplied by quartz-fibre optics to an array of 24 individual detectors. The operating principle is shown in Figure 1. A fixed arrangement of the sample cups, cuvettes and dispensing tips is used. Reaction mixtures are introduced into the photometer in thermostated aluminium blocks of 24 cuvettes, in which they are also prepared and preincubated using a range of additional system modules – parallel dispenser 216, incubator 354, mixer 369 and centrifuge. The block is manually transferred between modules. Dispensing order, volumes and incubation times may be selected according to the requirements of a particular assay method.

The 216 dispenser is a parallel dispenser for 24 samples or reagents. The reagents are poured into each cup, which may be filled in advance and transferred to the dispenser as needed. When dispensing, a disposable tip is used for each sample to eliminate cross contamination or carry over between the samples. Aspiration of a sample from a specimen can also be performed manually using a Multipipette which is a four-channel hand-pipette especially designed for the system cuvette blocks, or by using a semi-automatic diluter (eg the Micromedic automatic pipette model 25002, Micromedic System Ins, Philadelphia, USA).

The reaction temperature is maintained with an accuracy of ± 0.1°C of the set temperature and a between variation of ± 0.05°C by heating circuits in the block. The temperature of

```

CREATININE        UMOL/L

1  METHOD NUMBER  6
2  EP(1), KIN(2)  2
3  WAVELENGTH(NM) 510
4  FILTER NUMBER  6
5  MEAS TIME(MIN)  1
6  INCR(1),DECR(2)  1
7  TEMPERATURE(C)  37
8  STD RQD(1),NOT(2) 1
9  BLK(1),SER(2) STD 1
10 NUM OF STD PAIRS 2
11 FIRST CONC  0
12 STD CONC STEP 88.40
13 A (CONC/ABS) 1.000
14 C (ABS)  0
15 MIN A0 (ABS)  0
16 MAX A0 (ABS)  2.000

13.27.06
79-01-09
```

*Figure 2. Listing of all parameters used for the test.*

the cuvette block can be checked before the measurement by pressing the CUVETTE TEMP key on the control module.

The analyser has been programmed for ten different equilibrium or kinetic methods (selected by the user), each of which is characterised by 16 different variable parameters. A list of parameters is shown in Figure 2. This includes the measuring mode, whether the measurement is kinetic or equilibrium, the wavelength, the temperature (25, 30, 35 or 37°C), the measurement time for kinetic methods (1 to 10 min variable in steps of 1 min), the direction of the kinetic reaction, the number and concentration of standards, possible factors used in calculating the results, and the minimum and maximum allowed limits for a kinetic reaction. Because these parameters are variable, the user is not bound to ten methods.

The changeover from one method to another or the modification of the parameters is performed through the keyboard. This is shown in Figure 3. To change the method the METHOD NUMBER key, the desired numeric key and the ACCEPT key are pressed. The parameters of the newly selected method are then valid. To alter the value of a parameter the PARAM NUMBER key, the number of the desired parameter and the ACCEPT key are pressed followed by the new value and the ACCEPT key. All the altered parameters are valid until the power is switched off. The LIST function displays the valid parameters for checking.

Standards, if use, are analysed simultaneously with the samples. The calibration curve is calculated from these standard samples using a straight line fit. In equilibrium methods the concentration of the samples are calculated with the aid of factors which result from the calibration or which are entered by the user through the keyboard (maximal factor 9999). In kinetic methods 12 absorbance readings are measured for each standard and sample (the first readings can be made within 30 seconds after initiation of the reaction). The rates (dA/min) are calculated using a straight line fit. The concentrations are calculated as explained above.

Very recently Kone Oy, Instrument Division has introduced a parallel 8-channel sample processor "D", which performs the dispensing, mixing and incubation of samples and reagents completely automatically in the cuvette block.

### Results and discussion

### Rate of analyses

It was possible to analyse 24 samples (one block containing usually four standard and 20 serum samples) in 10 min for albumin, 35 min for alpha-amylase and 25 min for the other methods with incubation periods included. If the pipetting of the following blocks was started during the incubation period of the first block, 2-3 more blocks could be assayed in about 15 min for all the analyses tested.

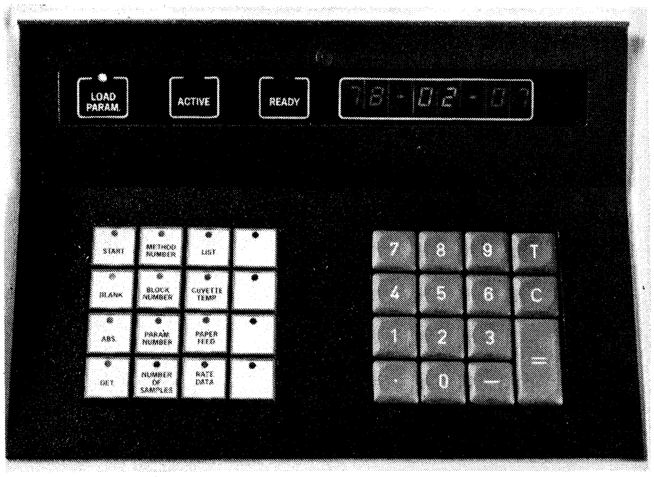

*Figure 3. The keyboard of the C Parallel Analyser.*

Table 1. Within-day and day-to-day precision (n = 20) of the tests on the C Parallel Analyser.

| Test | Within-day | | | | | | Day-to-day | |
|---|---|---|---|---|---|---|---|---|
| | Low mean | CV% | Medium mean | CV% | High mean | CV% | Medium mean | CV% |
| ALP, U/l 37°C | 80.6 | 2.6 | 195.4 | 1.9 | 330.8 | 1.5 | 188.3 | 2.8 |
| AST, U/l 37°C | 14.0 | 2.5 | 40.6 | 1.7 | 226.3 | 1.7 | 35.2 | 4.9 |
| Amylase, U/l 37°C | 82.2 | 2.3 | 177.1 | 2.1 | 946.5 | 3.0 | 213.3 | 4.7 |
| Albumin, g/l | 27.2 | 1.6 | 38.9 | 1.7 | 47.3 | 1.0 | 39.5 | 1.6 |
| Cholesterol, mmol/l | 3.54 | 1.7 | 5.55 | 0.9 | 10.1 | 0.9 | 5.62 | 2.1 |
| Creatinine, µmol/l | 44.4 | 2.5 | 84.8 | 2.5 | 702.7 | 0.6 | 87.1 | 3.6 |
| Triglycerides, mmol/l | 0.81 | 2.7 | 1.62 | 2.4 | 4.52 | 2.3 | 1.61 | 4.4 |

Table 2. Within-day precision of the whole system when different types of pipettes were used in serum sample pipetting (n = 20).

| Test | Sample/reagent volume (µl) | Micromedic pipette | | Four channel hand pipette | |
|---|---|---|---|---|---|
| | | mean | CV% | mean | CV% |
| Albumin, g/l | 10/1400 | 38.9 | 1.7 | 39.3 | 5.6 |
| Total protein*, g/l | 10/1000 | 69.8 | 1.0 | 74.1 | 11.3 |
| " " | 20/2000 | 70.6 | 0.5 | 71.8 | 2.8 |
| " " | 50/5000 | 70.9 | 0.4 | 70.7 | 1.3 |
| AST, U/l 37°C | 150/1100 | 24.3 | 2.4 | 23.1 | 2.7 |

* Biuret method – Mercotest no 3327 (E Meck AB).

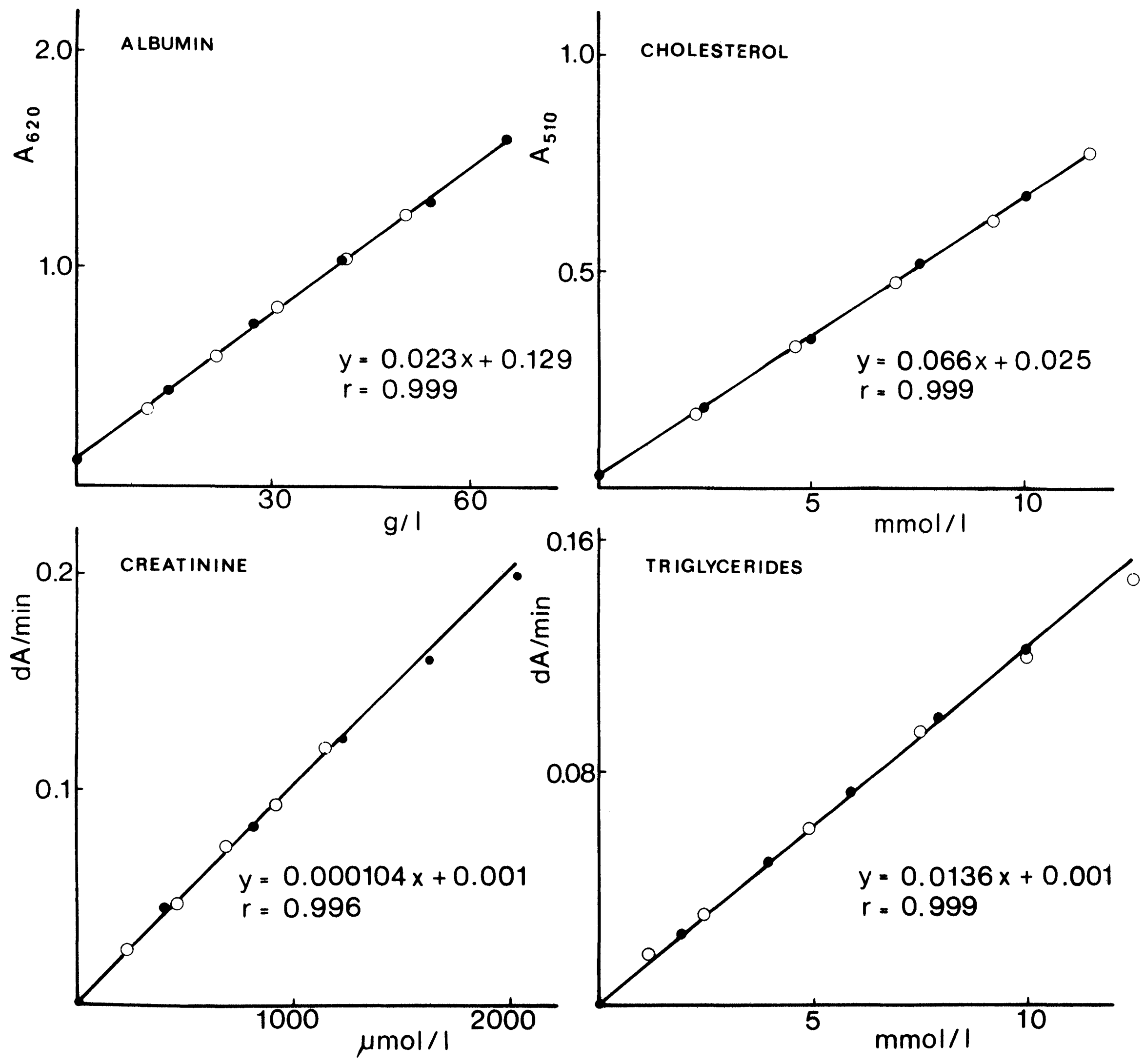

*Figure 4. Linearity of albumin, cholesterol, creatinine and triglycerides tests on the C Parallel Analyser, determined using dilutions of aqueous standards and patient sera with high concentration (o).*

### Precision

The within-day precision was evaluated by analysing specimens of sera at three different levels of concentration or activity of analytes (low, medium and high) (n = 20), and day-to-day precision at one concentration or activity of analytes (n = 20). The mean values and variation coefficients are given in Table 1. Both within-day and day-to-day precisions were good, generally less than 3.0% and 5.0%, respectively.

A Micromedic diluter or a 24-channel dispenser were used in sample pipetting. The Micromedic diluter was always used for serum volumes below 50μl, because earlier experience indicated that the smallest serum volume which can be reliably dispensed with the 24-channel dispenser is 50μl [6].

The suitability of the four-channel hand-pipette in serum sample pipetting was also tested. The pipette gave acceptable precision above 50μl, but under this volume it was found to be more reliable to use a diluter (Table 2).

The new parallel 8-channel sample processor "D" was also evaluated. From preliminary results on this machine the precision within blocks (20 different blocks were tested) was 1.2% and 1.0%, when 10 and 50μl volumes of p-nitrophenol in serum plus 550μl of buffer were pipetted and the absorbances were measured at 405 nm. This indicates that problems in the pipetting of small sample volumes for parallel analysers can probably be solved using this instrument.

### Linear range

Linearity of the methods was determined using aqueous standards and specimens containing high concentrations or activities of the various constituents. These were diluted with 0.9% NaCl before analysis to cover a range of values. The range of linearity for ALP and AST was found to be at least up to ten times the upper limit of the normal reference range (not shown), for alpha-amylase up to 1000 U/l (not shown), for albumin at least up to 65 g/l (Figure 4), for cholesterol at least up to 11.0 mmol/l (Figure 4), for creatinine up to 1500μmol/l (Figure 4) and for triglycerides up to 9.0 mmol/l (Figure 4). These ranges were sufficiently extensive to allow most specimens to be analysed without dilution. In addition, Figure 4 shows the sensitivity of the methods.

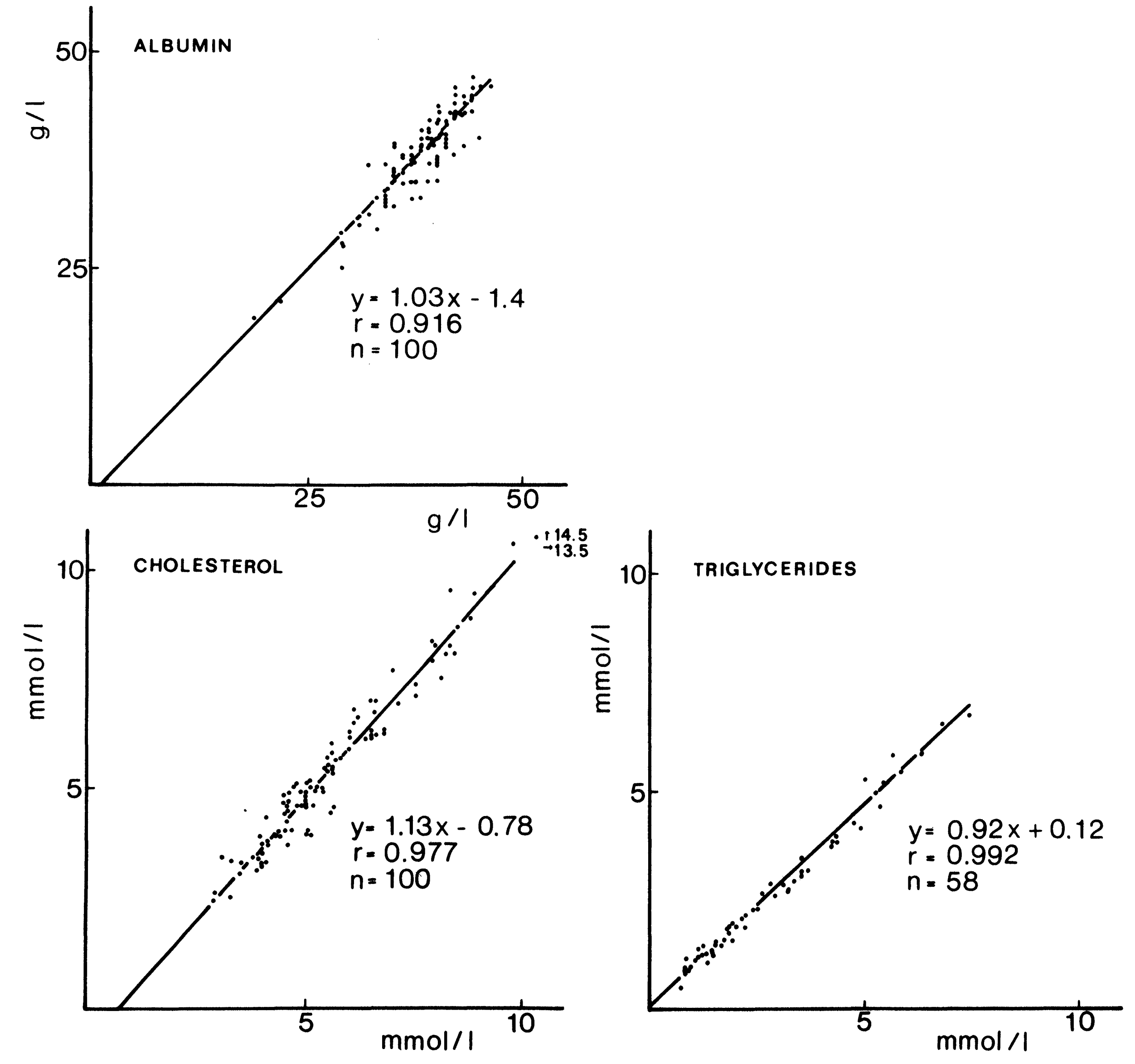

*Figure 5a (above) and 5b (opposite). Comparison of patient values between the methods used routinely in the authors' laboratory (x) and in the C Parallel Analyser (y).*

**Comparison of patient sera**

Analyte concentrations or activities obtained by the C analyser methods were compared with the values obtained from the procedures routinely used in the authors laboratory (except for alpha-amylase). All such comparisons were made on the same day within ½-3 hours of each other, 100 patient sera being used for each comparison (58 for triglycerides). A summary of the correlation and regression data is given in Figure 5. Correlation coefficients obtained were 0.916 for albumin values, 0.977 for cholesterol values and over 0.99 for the others.

The poor correlation obtained for albumin values probably reflects a variation caused by the reagents and/or the reaction conditions (cf work by Spencer and Price [7]) rather than instrument variability. The albumin values obtained from the procedure used in the Technicon SMAC do not compare well, with values obtained by the more specific methods [8].

In ALP and AST studies, for which the same methods were used on both the LKB reaction rate analyser and the C analyser, slopes close to 1.00 were obtained. A slight difference in the slope for ALP could not be due to a variation in the optical characteristics of the filters of the two instruments, because 405 nm filters were calibrated using solutions of p-nitrophenol of known absorbance. Several other explanations are possible as different types of pipette and different measurement time intervals were used. In addition, the primary data were analysed differently.

When using the C analyser for alpha-amylase determinations it was not possible to use a computer to obtain results as in a bigger parallel analyser, the System Olli 3000 [4], which offers the more complicated programs for non-linear calibration curves. The results were consequently calculated manually with the aid of the Pharmacia standard curve. Higher results obtained with the C analyser can be explained both by differences between different batches of the substrate and too high an incubation temperature for the reactions. The temperature of the incubator 354 is 37.5 - 37.6°C because it has been designed especially for the pre-incubation of the kinetic analysis [1].

In view of the different methods and the different standards used, the correlation data for cholesterol (enzymatic/non-enzymatic method), creatinine (kinetic/equilibrium method) and triglycerides (kinetic/equilibrium method) were considered acceptable. In the kinetic method for the determination of triglycerides especially, the control of the reaction temperature and the exact timing of the measurements were necessary to obtain accuracy and precision.

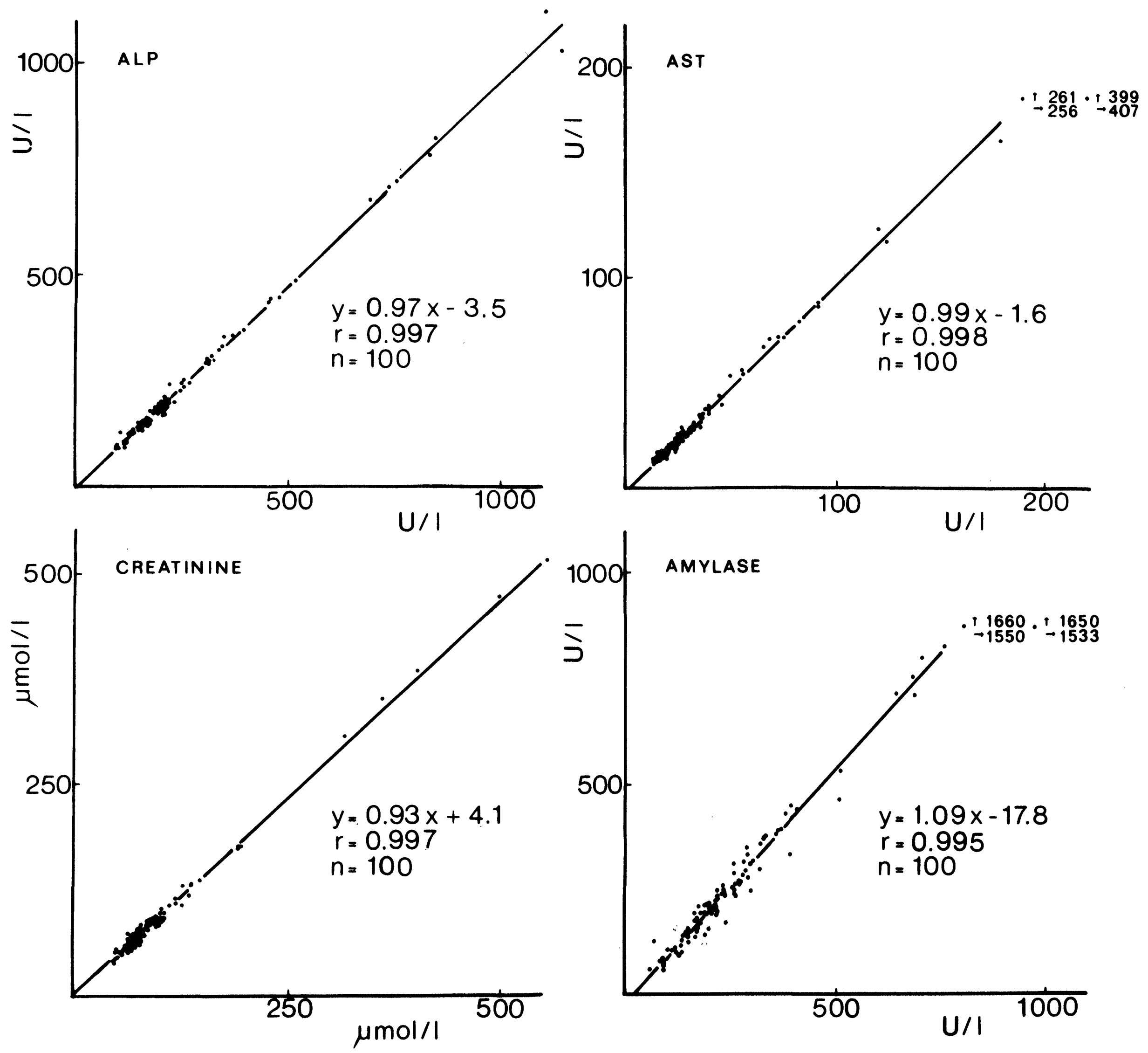

*Figure 5b*

**Error messages**

The error detection system in the C analyser adds validity to the answers. A series of messages are used which indicate whether or not there are errors in calibration or in the reaction temperature, that the initial absorbance of the kinetic reaction is too high or too low, that the reaction rate curves fail to satisfy the linearity criteria etc. However, the linearity error code is printed only if the linearity error is greater than 20%. The acceptability limits of linearity should be smaller and/or the operator should be able to select a value for them. The graphical print-out of kinetic measurements is not possible in the C analyser, as it is in the System Olli 3000 analyser. However, if the error message indicates the nonlinear reaction, the user can check how the reaction is proceeding by using RATE DATA key. Rate data consists of (1) four abosrbance values, which are mean values of three successive readings, (2) three dA/min values obtained from the successive mean absorbances respectively, and (3) results in concentration units, which are calculated for each dA/min. The error messages are not included in the programs of the System Olli 3000 analyser.

**Reliability**

The primary purpose of this evaluation was to test the suitability of the C Parallel Analyser for the laboratories in the district of Oulu University Central Hospital. During the two months of the evaluation period no service was needed. After the analyser had been in Oulu City Hospital for one year, the downtime was three days, one due to the change of the UV-filter, and two due to the repair of the ignition unit for the Xenon-lamp. Additionally, the timer of the preincubator was broken, but it did not hold up work. In Oulu City Hospital the analyser is used for five days weekly and about 300 analyses are performed daily.

### Conclusion

The C Parallel Analyser has proved to be an instrument with good reliability and precision. Advantages were the speed of analysis, the ability to change chemistries easily and quickly, versatility in accommodating kinetic and equilibrium tests equally well, and the ease with which methods can be modified. The ease of operation is impressive and the operator can learn the keyboard manipulations rapidly. Errors and malfunctions are indicated through a thermal printer. Because standards and samples can be analysed in parallel, the C analyser has much potential especially in the kinetic analyses of serum nonenzymatic constituents. However, because the first readings can be made only within 30 seconds after initiating the reaction, analyses of very rapid kinetic reactions, for instance kinetic turbidimetric assays of serum immunoglobulins are difficult. The C analyser also lacks a graphic printer.

A semi-automatic diluter (or the D parallel sample processor) is needed for the pipetting of small sample volumes. The analyser should have an application in performing a range of analyses in laboratories where around 150-600 daily analyses are performed and it is also suitable for the analysis of emergency samples.

ACKNOWLEDGEMENTS

The loan of the analyser used in this investigation was arranged by Kone Oy, Instrument Division. Marjatta Leppilampi, MSc, is thanked for excellent technical assistance and Maija-Leena Kallio, MSc, for providing information on the reliability of the instrument.

REFERENCES

[1] Adlercreutz, H., Peltonen, V., and Voipio, T., *Clinical Chemistry,* 1975, **21**, 676.

[2] Puukka, R. and Puukka, M. Third European Congress of Clinical Chemistry, Brighton, England, June 3-8, *Abstract book,* 1979, p. 44.

[3] Committee on Enzymes of the Scandinavian Society for Clinical Chemistry and Clinical Physiology. *Scandinavian Journal of Clinical Laboratory Investigations,* 1974, **33**, 291.

[4] Puukka, R. *Scandinavian Journal of Clinical Laboratory Investigations,* 1978, **38**, 127.

[5] Puukka, R., Jokela, H. and Puukka, M. *Scandinavian Journal of Clinical Laboratory Investigations,* 1978, **38**, 189.

[6] Hammond, G.L., Leinonen, P. and Vihko, R. *Clinical Chemistry,* 1979, **25**, 129.

[7] Spencer, K. and Price, C.P. *Annals of Clinical Biochemistry,* 1977, **14**, 105.

[8] Cederblad, G., Hickey, B.E., Hollender, A., and Åkerlund, G. *Clinical Chemistry,* 1978, **24**, 1191.

### Erratum

*The Journal of Automatic Chemistry,* April 1980, 2, 2, 66-75.

**A compact automated microprocessor-based flow analyser**

**Michael A. Koupparis, Ken M. Walczak and Howard V. Malmstadt**

The authors have asked that the following errors in the above paper be brought to the attention of the readers.

On p71, the second sentence of the fourth paragraph should read, "The 1 to 0 transition was chosen as the power-on state of the parallel I/O ports to ensure that the control signals were not activated when power was applied to the microcomputer or a reset sequence was initiated."

On p71, the final paragraph should state, "The absorbance measurements of Table 2 were carried out at 520 mm."

On p73, in the first complete paragraph, the sixth and seventh sentences should read, "The minimum volume required to move the old solution from the cell (dead volume of mixer, flow cell, and their connections) was found to be 100 μl. 100-25 μl can be chosen by adjusting the mechanical stoppers of the automatic pipetter."

On p74, the ascorbic acid standards of the text and in Table 5 were made of 5-40 mg/l in 0.05M oxalic acid solution.

On p75, in the table of References, the fifth reference was published in 1977.

On p74, the slope in Table 5 should read −0.02366.

